# Current Uses for Medical Imaging With Orthopaedic Implant Technology

**DOI:** 10.1002/jor.70142

**Published:** 2026-01-15

**Authors:** Jordan S. Broberg, Matthew G. Teeter

**Affiliations:** ^1^ Department of Orthopaedics, Faculty of Medicine University of British Columbia Vancouver British Columbia Canada; ^2^ Bone and Joint Institute Western University London Ontario Canada

## Abstract

Musculoskeletal applications have been at the forefront of medical imaging since the first‐ever x‐ray was taken of a hand. Radiography remains routine in orthopaedics for surgical planning and diagnosing implant complications. Fluoroscopy is used to guide intra‐operative implant placement and to assess kinematics of different implants. Computed tomography (CT) is used to develop surgical robotics plans and patient‐specific instruments, while magnetic resonance imaging (MRI) can diagnose soft tissue reactions near implants. Ultrasound is less frequently used in orthopaedics but can provide assessments on tendons and ligaments as well as diagnose tissue pathologies. Additional modalities are becoming available that show promise for orthopaedic implant applications, including weight‐bearing CT (WBCT), dynamic or four‐dimensional CT (4DCT), low field strength (0.55 T) MRI, and positron emission tomography (PET). However, hardware and software costs, scanner accessibility, and high burden for the analyses has limited many of the more advanced imaging techniques to research centres or tertiary care facilities. Across all modalities, computational modelling and artificial intelligence applied to medical images allow more complex uses of imaging data. Time‐consuming segmentation tasks can be automated, which allows patient‐specific finite element modelling and other types of simulations to be performed more routinely for surgical planning and implant evaluation. Additionally, radiomics can be applied to images to identify implant models and improve diagnoses of implant complications. Metal artifacts near implants are also being reduced by new reconstruction algorithms. Thanks to innovations in medical imaging technology, the care of patients with orthopaedic implants is being improved by enabling more patient‐specific interventions.

## 1 Introduction

**Figure 1:**
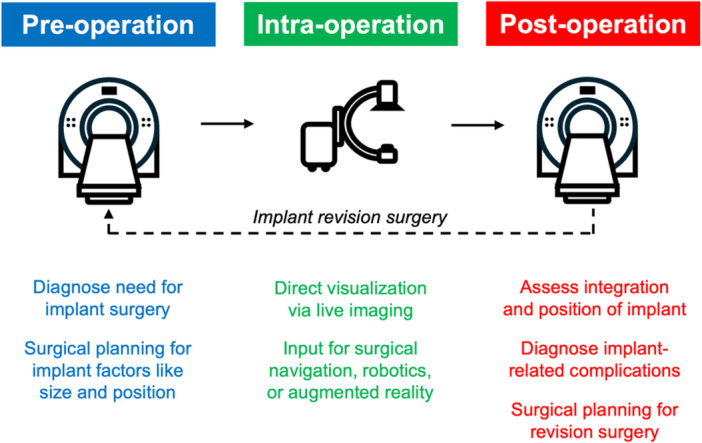
Schematic showing the clinical uses of medical imaging for patients requiring an orthopaedic implant.

The birth of orthopaedic imaging is aligned to Wilhelm Roentgen's discovery of x‐rays, when he placed his fingers within the x‐ray beam and recognized the resulting image on the film screen [1]. John Charnley, the pioneer of total hip arthroplasty – the operation of the century [2] – was also a pioneer in the use of radiographs for pre‐operative surgical planning and diagnosing implant‐related complications [3, 4]. Today, a variety of medical imaging modalities are used in research and clinical practice relating to orthopaedic implants (Figure 1). Each modality comes with advantages and disadvantages, including factors like cost, ionizing radiation dose, differences in tissue contrast, and weight‐bearing status, all of which are highly relevant to orthopaedic implant applications. Therefore, the intended application must be carefully considered when choosing an imaging modality to implement. The intention of this review is to provide a brief overview of the imaging modalities and associated digital technologies that are being implemented with orthopaedic implants. We discuss traditional modalities first before covering modalities less commonly applied to orthopaedics. Within each section the current applications and emerging technologies of each modality are summarized. We finish with a discussion on new trends and existing limitations in the field of orthopaedic imaging.

### 1.1 Radiography and Fluoroscopy

Radiography (plain x‐rays) is the oldest medical imaging modality but remains highly relevant to modern orthopaedic implant technology. Inexpensive and fast to acquire, radiographs are commonly used by orthopaedic surgeons for diagnosing pathology and for surgical planning [5]. Radiographs are particularly useful because they can be acquired with the subject in weight‐bearing position [6]. Current hot topics with orthopaedic implant technology like personalized implant position for total hip and knee arthroplasty rely heavily on radiographs to measure hip‐knee‐ankle angle for knees and spinopelvic position for hips [7, 8, 9].

Since radiography systems are quite mature, most innovation is in software for image processing and analysis, particularly leveraging artificial intelligence. Applications of artificial intelligence for orthopaedic implant radiography include implant identification, limb alignment measurements, osteoporosis screening, and identifying complications like aseptic loosening or fractures [10]. This speeds up and makes more repeatable tasks like alignment measurements and helps surgeons and radiologists to identify subtle features on images to improve diagnostic accuracy. However, the “black box” nature of many artificial intelligence algorithms diminishes clinicians' trust. [11] Therefore, developers of this technology should also use large, multi‐institutional and/or open‐source annotated data sets to externally validate their algorithms and increase the trust in an algorithm's generalizability. ^12^ Developers should also explore explainable artificial intelligence, a subfield dedicated to developing artificial intelligence algorithms that provide interpretable rational for their decisions and actions in order to increase user trust and encourage clinical implementation of their algorithms [12].

A specialized implementation of radiography used in orthopaedic implant research is radiostereometric analysis (RSA). RSA uses synchronized stereo x‐rays and specialized calibration hardware to track the migration of implants relative to ~1 mm Tantalum marker beads inserted into the bone surrounding the implant during surgery [13]. A large amount of migration during the first months to years following arthroplasty surgery is highly predictive of later aseptic loosening failure, with specific thresholds for acceptable migration being known for various types of implants [14, 15, 16, 17]. RSA can also be used without marker beads to measure implant wear and joint kinematics [18, 19]. Given the need for specific hardware and consent to insert bone marker beads, RSA has seen limited use in only a handful of institutions [20]. However, computed tomography‐based RSA has recently become available, and will likely replace traditional RSA for research to assess implant safety and for clinical diagnosis of implant loosening via inducible displacement exams [21]. While CT‐RSA may expand the number of institutions able to perform RSA studies, widespread adoption will depend on overcoming the significant burden of image analysis.

Another radiographic imaging technology being used in orthopaedic implant research is the biplanar fan‐beam X‐ray system, often referred to as EOS [22]. These systems consist of a pair orthogonally arranged X‐ray tubes and corresponding slot‐scanning detectors that move vertically to simultaneously acquire two radiographs of the whole body, or a particular region of interest. Advantages of EOS include their lower radiation exposure, their ability to scan the whole body in weightbearing positions, and their ability to generate 3D reconstructions from the two simultaneously acquired 2D radiographs [23]. Common uses in orthopaedic implant research include pre‐ and post‐operative measurement of spinopelvic alignment, hip‐knee‐ankle angle, lower limb torsion, and coronal plane alignment of the knee [24, 25, 26, 27].

Fluoroscopy (or fluoro) is another specialized implementation of radiography taking video x‐rays in either a single plane or stereo setup. Fluoroscopy is often used by surgeons intra‐operatively to guide implant placement for spinal fusion or total hip arthroplasty [28, 29]. Surgical navigation software has been developed that provides implant positioning guidance based on patient characteristics and surgeon preferences [30]. In research, fluoroscopy has been used to determine joint kinematics and compare motion profiles between different types of implants and surgical techniques across multiple joints [31, 32, 33, 34]. Here again, artificial intelligence is being applied to segment images and register implant models to automatically perform kinematic measurements [35, 36]. Work is underway to bring fluoroscopic exams of kinematics to the clinic for surgical planning and evaluation [37, 38].

### 1.2 Computed Tomography

Following radiography, computed tomography (CT) is the second‐most used imaging modality with orthopaedic implants. CT uses multiple radiographic projections around the subject to generate multi‐planar reconstructions and 3D renderings of patient anatomy. This offers substantial advantages for measuring alignment, determining the relative positions of anatomical features, and surgical planning, but comes at a cost of increased ionizing radiation compared to plain radiographs [39, 40, 41]. However, improved reconstruction algorithms, particularly those implementing artificial intelligence, have enabled a reduction in radiation dose through techniques like low‐dose or sparse‐view CT, all while still providing good quality images for orthopaedic applications [21, 42, 43, 44, 45]. Similarly, the presence of metal implants in CT exams produces metal artifacts, but the magnitude of these artifacts have been reduced using new metal artifact reduction exam protocols and reconstruction algorithms [44, 46].

Bone shape and density can be assessed from CT scans, which is useful for the design of implants. Statistical shape models can be generated from libraries of CT scans and used to determine appropriate implant sizing [47, 48, 49]. Similarly, statistical density models can be used to determine the appropriate placement of implant fixation features to maximize stability and encourage bone ingrowth [50, 51]. Large databases of CT scans help facilitate implant design based on factors like sex and ethnicity [52, 53]. CT scans are frequently used as inputs to finite element models of implants in bone, where different scenarios can be modelled and validated against biomechanical tests with cadaveric or synthetic bone [54, 55, 56].

In recent years, CT scans have become common to design patient‐specific surgical guides and patient‐specific implants suitable for production using additive manufacturing [57, 58, 59, 60]. CT scans are also used for image‐based surgical navigation and robotics systems [39, 41, 61, 62]. They provide critical information regarding limb alignment and implant positioning. Using CT scans for surgical planning may be particularly useful in cases where implant insertion is required around existing implanted hardware or if bone defects are expected, such as in revision scenarios [49, 63]. CT scans for surgical planning typically have standardized scan parameters and occasionally require the addition of devices such as a motion rod so that patient motion can be detected and corrected [64]. The standardized nature of these scans lends itself to automated segmentation of landmarks and geometry. Other information that can be extracted from the CT scan like bone density has so far been less commonly used in surgical planning but offers potential to aid in decision making to determine the appropriateness of cemented versus cementless implants, for example [65].

CT scans are also useful for implant evaluation post‐operation. Measurements of implant position using CT scans have been correlated to patient outcomes and dissatisfaction [66]. CT can be used to measure polyethylene wear and identify osteolysis, as well as other bone defects [49, 67, 68]. CT‐based RSA is in the process of replacing conventional model‐based RSA to measure implant migration over time as well as for inducible displacement exams to diagnose implant loosening [13, 69].

**Figure 2:**
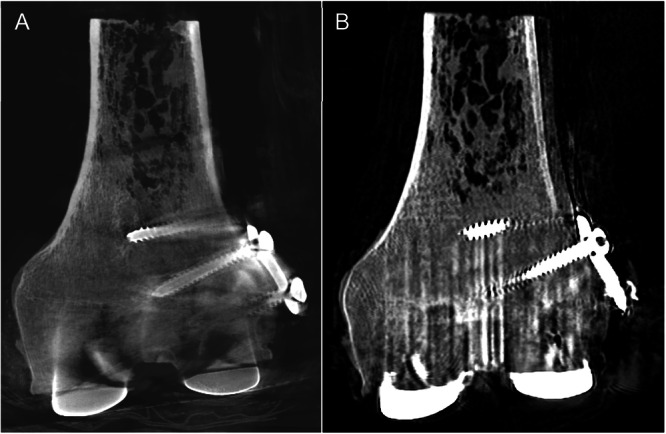
CT scans of a distal femur with periprosthetic fracture fixation plate and screws adjacent to a femoral component from total knee arthroplasty, acquired using a weight‐bearing CT scanner (A) and conventional CT scanner (B).

Hardware developments in CT scanner technology will offer new information that might make surgical planning even more impactful (Table 1). Weight‐bearing CT scanners are cone‐beam scanners that, unlike conventional CT scanners, have the advantage of being oriented to acquire exams with the subject standing in a position where the joint is loaded [70]. While the first systems to be marketed were limited to acquisitions of the foot, ankle, or knee, current technology can now scan up to the hip. New weight‐bearing CT scanners are coming to market that will scan from the foot to the cervical spine. This is particularly exciting to understand the interaction of loading between body segments as it relates to various implants, such as the spinopelvic region in total hip arthroplasty and the lower limb in total knee arthroplasty. Evidence from paired unloaded and loaded weight‐bearing CT scans show that acquiring images in a loaded position can substantially change the interpretation of joint space narrowing and anatomical position [71]. Weight‐bearing CT systems are already being used to plan and evaluate total ankle and total knee arthroplasty [72, 73]. They are also being used to perform inducible displacement exams for CT‐RSA to assess implant fixation [74]. Additional benefits of weight‐bearing CT scanners are that they are smaller and less costly than conventional CT scanners, and given their cone‐beam scanner design they impart significantly less ionizing radiation [75]. They may show different metal artifacts around implants than conventional CT scanners (Figure 2).

**Table 1:** Comparison of the main CT modalities across key parameters.

CT modalities	Dose	Resolution	Motion capability	Artifact suppression	Cost
Traditional CT	+++	++	No	++	++
WBCT	+	+++	No	++++	+
4DCT	++++	+	Yes	+	+++
PCCT	++	++++	No	+++	++++

Dynamic joint motion can now be studied using four‐dimension CT scans (4DCT). Offered on certain newer CT scanners, 4DCT was originally developed for radiation oncology and cardiac applications, but there are clear advantages for orthopaedics and the technology has now been applied to acquire 3D CT scans as subjects move their joints [76]. With 4DCT, natural joint motion and the interaction of bones within joints can be studied and are particularly helpful in regions of complex anatomy, such as the wrist or patellofemoral joint [77, 78]. The trade‐off with 4DCT is higher ionizing radiation than static 3D scans, as well as the potential of motion artifact [76]. As a work‐around to reduce radiation dose it is possible to acquire a single high resolution (and high ionizing radiation dose) static CT scan followed by low‐dose, lower resolution 4DCT scans, then register a 3D model from the high‐resolution scan to the low dose scans [79].

Another development in CT scanner hardware is photon‐counting CT. The benefits of photon‐counting CT scanners are better dose efficiency, reduced noise, and improved spatial resolution [80]. Since each photon is counted and sorted by its energy, photon‐counting CT can perform spectral decomposition and spectral reconstruction, providing enhanced contrast between tissue types [81]. One application of photon‐counting CT in relation to orthopaedic implants is a detailed visualization of cortical and trabecular bone and the assessment of bone quality as opposed to simply bone density [81]. Additionally, metal artifacts around implants can be greatly reduced with photon‐counting CT, allowing fixation and osseointegration to be studied closer to the implant [81, 82]. However, photon‐counting CT is still in its early stages. For it to be more widely adopted, greater availability of scanners and more technical advancements are needed.

### 1.3 Magnetic Resonance Imaging

Musculoskeletal applications of MRI mostly take advantage of its excellent soft tissue contrast to evaluate cartilage and ligaments. MRI has no ionizing radiation, which is a substantial benefit over CT. However, MRI scanning of certain implants, particularly at ultra high field strengths, may not be appropriate given the potential for implant displacement and damage to surrounding tissue from heating [5]. The presence of metallic implants also introduces significant artifacts into MRI scans [83]. As these susceptibility artifacts are proportional to the strength of the main magnetic field (B_0_), there is benefit to scanning at lower field strengths; however, there are advanced MRI sequences for reducing artifacts that make it feasible to image implants at higher field strengths [84, 85].

MRI can be used for surgical planning and in some instances the ability of MRI [86] to visualize tissue like cartilage can be advantageous, for example to produce patient specific surgical guides that result in fewer surgical outliers than guides produced from CT [87]. MRI may also be useful to determine whether unicompartmental or total knee arthroplasty is indicated from cartilage assessment, or whether total or reverse shoulder arthroplasty is indicated based on rotator cuff integrity [88, 89]. MRI also helps clinicians with patient management decisions. For example, MR findings of infectious synovitis could highlight the need for repeated laboratory tests in cases where periprosthetic infection is suspected but test results are inconclusive [90]. MRI can also aid in the decision to proceed with arthroscopic debridement in cases of patellar clunk syndrome [91], and can help diagnose recurrent hemarthrosis after total knee arthroplasty and unsatisfactory conservative management [92].

The physics of MRI dictate that metal artifacts will be worse at greater field strengths. A recent innovation in MRI hardware is low field MRI (e.g. 0.55 T) scanners. These scanners are cheaper to produce and operate than 1.5 T scanners and offer increased patient comfort via low noise and wide or open bores [85]. Metal artifacts adjacent to total hip implants are reduced with 0.55 T scanners compared to 1.5 T and 3 T [84, 85]. Although 0.55 T scanners are scarce and are only beginning to be studied for orthopaedic applications, they have already been shown to have high specificity for diagnosing periprosthetic joint infections [93].

**Figure 3:**
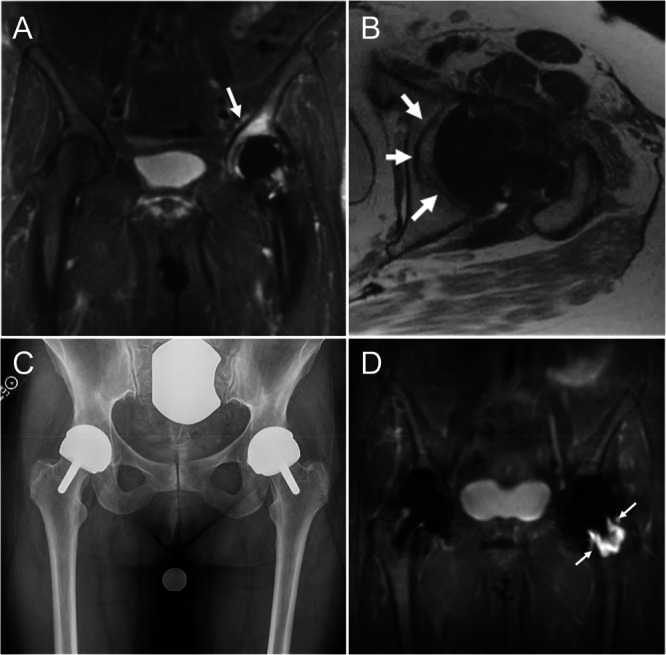
Examples of the utility of 1.5 T MRI with susceptibility artifact reducing sequencies for evaluating hip arthroplasty. (A) Coronal fat suppressed multispectral MR image in a 56‐year‐old woman one year status post left arthroplasty demonstrates a severe stress reaction in the dome (arrow). (B) Axial fast spin echo image in the same patient demonstrates a complete lack of osseous integration of the acetabular component (arrows). (C) AP radiograph view of the pelvis of 56‐year‐old woman 7 years following bilateral resurfacing arthroplasty with left hip pain demonstrates minimal radiolucency adjacent to the femoral stem. (D) Coronal inversion recovery multispectral MR imaging demonstrates extensive femoral neck osteolysis (arrows) not discerned on the radiograph. Images courtesy of Dr. Hollis Potter, Hospital for Special Surgery.

The more widely accessible 1.5 T and 3 T MRI scanners can still image tissues adjacent to orthopaedic implants thanks to the development of advanced MRI pulse sequences for reducing susceptibility artifacts (Figure 3) [86]. The two common metal artifact reduction methods include spectral binning and spatial encoding [94], which both have been shown to reduce image distortions by roughly a factor of 10 when compared to standard fast spin echo images, but come with the drawback of image acquisition time increasing by approximately a factor of 1.5 to 2. A third hybrid technique combining the strengths of both methods is now also available for use by both clinicians and researchers. These artifact reduction methods have made MRI particularly useful for identifying adverse local tissue reactions around metal‐on‐metal hip implants and other implants with excessive wear and corrosion [95]. Other implant‐related complications like arthrofibrosis and periprosthetic joint infection can also be diagnosed using MRI [96, 97]. MRI can also be used to study bone‐implant muscle properties following arthroplasty [98, 99]. Additionally, tissue properties can be quantified via techniques such as T2 mapping, including near orthopaedic implants [100]. Ongoing developments in deep learning reconstruction techniques to augment these metal artifact reduction sequences will help reduce the scan time and improve the resolution and quantitative capability of periprosthetic imaging [101, 102].

Developments in MRI pulse sequences have made other advances in addition to reducing metal artifacts. The development of ultrashort echo time (UTE) and zero echo time (ZTE) sequences allow MRI to provide CT‐like visualization of bone prior to arthroplasty, allowing for analysis of bone microstructure and assessment of many joint pathologies, such as early osteophyte formation or femoroacetabular impingement [103]. Paired with its excellent tissue contrast, this could make MRI an “all in one” tool for examining both bone and soft tissue via multiparametric image acquisitions that eliminates ionizing radiation in the planning stages before surgery. These sequences also detect enough signal for direct quantitative analysis in other tissues with low transverse relaxation times, like tendons, and are useful in applications such as detecting soft tissue calcifications and assessing hip joint width prior to arthroplasty. When combined with a metal artifact reduction sequence, the UTE sequence also improved visualization of short T2 musculoskeletal tissues (e.g. tendons, ligaments, and cortical bone) in the presence of a joint replacement when compared to the standard metal artifact reduction sequence, which are based on fast spin echo sequences [104].

### 1.4 Ultrasound

Ultrasound is advantageous as it does not impart ionizing radiation (unlike radiographs or CT) and is also much less expensive than MRI. This gives it great potential for use in rural or ambulatory surgical centres. However, ultrasound has seen comparatively less use for implant applications than these other imaging modalities. Due to the high acoustic impedance of bone and metal, conventional ultrasound waves are largely reflected at their surfaces, limiting direct visualization beyond these materials. This [5] has limited ultrasound mostly to the assessment of soft tissues like tendons and ligaments [105, 106], or diagnosing tissue pathologies like synovitis [107], adverse local tissue reactions [108], or periprosthetic joint infections [109]. However, techniques like shear wave elastography, axial transmission ultrasound, and quantitative ultrasound have been used in bone health and stiffness assessments, and to monitor fracture healing [110, 111, 112, 113]. Ultrasound can also be applied to generate 3D bone models, which can then be used to pre‐operatively template implant size or integrate with surgical navigation or robotics systems in total knee arthroplasty [114]. Ultrasound‐based navigation is also being investigated for surgical navigation of pedicle screw placement in spinal fusion surgery [115].

### 1.5 Nuclear Medicine

Nuclear medicine modalities such as bone scintigraphy (bone scans) are well established in the diagnosis of orthopaedic implant complications like periprosthetic joint infection or aseptic loosening [116]. There has also been longstanding interest in the use of single photon emission computed tomography (SPECT) tracers like technetium‐99m (Tc99) and positron emission tomography (PET) tracers like 18F‐fludeoxyglucose (FDG) and 18F‐sodium fluoride (NaF) to serve similar diagnostic purposes in combination with CT or MRI scanners [117]. However, the expense of SPECT/CT, PET/CT, and PET/MRI along with less‐than‐ideal diagnostic sensitivity and specificity has prevented these modalities from becoming more popular, and they are currently not recommended for regular clinical use [118, 119]. Still, there remains excellent promise in these combined nuclear medicine modalities for arthroplasty applications [120]. Some of the biggest causes of implant failure are biological in nature, such as periprosthetic joint infection [121]. Ongoing pain after implant surgery is another common application that could benefit from nuclear medicine investigation, either of the joint or the brain [122].

**Figure 4:**
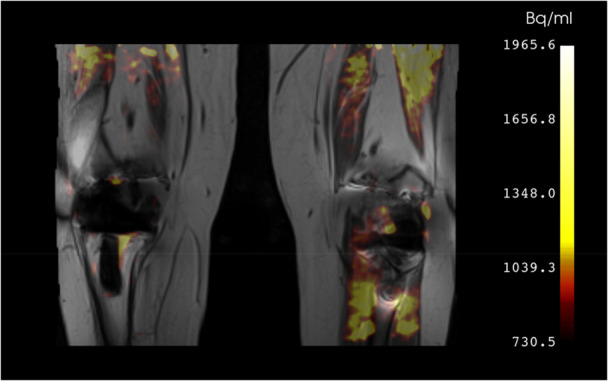
PET/MRI of a patient with bilateral total knee arthroplasty showing uptake of [18 F]FEPPA.

Promising developments for PET imaging of orthopaedic implants surround the generation of novel radiotracers (Table 2). Most studies have used FDG for generalized inflammation and NaF for bone turnover [123, 124, 125, 126]. New tracers have been developed for cellular targets like macrophages for inflammation (Figure 4) [127], fibroblasts for fibrosis [128], and glutamine for infectious biofilms [129]. These newer, more specific tracers may offer better insights into cellular activity for research studies and be more useful as a diagnostic tool for complications like infection, pain, or arthrofibrosis. Often, new tracers are developed for non‐orthopaedic applications (such as oncology) but are then re‐purposed to study similar cell types in bones and joints [130]. Theranostics, where diagnosis and therapy are combined, is at a very early stage for musculoskeletal health applications and is as yet unexplored for orthopaedic implants, but might be possible for applications such as diagnosing and treating infection [131, 132]. An exciting possibility is embedding theranostic capabilities directly into implants for early detection and treatment of complications like poor graft integration, infection, non‐union, or implant loosening [131].

**Table 2:** Summary of PET radiotracers.

**Tracers**	**Target**	**Stage of validation**	**Implant‐related use case**
FDG	Glucose metabolism	Used clinically	Inflammation, infection
NaF	Hydroxyapatite crystals	In clinical research	Bone turnover, bone metastases, fracture detection, arthritis severity
FEPPA	Macrophages	Early clinical research	Synovitis
FAPI	Fibroblasts	Early clinical research	Fibrosis
Glutamine	Infectious biofilms	Preclinical	Periprosthetic joint infection

In addition to better tracers, advanced analytical tools can improve the utility of nuclear medicine. Dynamic PET acquisitions allow tracer kinetics to be measured within joint compartments, enabling studies of metabolism and perfusion [133]. PET radiomics allow the pattern of tracer uptake to be measured alongside the traditional measures of uptake intensity so that tissue characteristics can be better understood [134].

### 1.6 Trends Across Imaging Modalities

Artificial intelligence is not only improving tasks like segmentation and recognizing pathologies, but is also enabling more complex computational modelling to be performed at a patient‐specific level. An emerging field that relies heavily on imaging is digital twins, which can use scans in combination with other patient data to predict outcomes [135]. Digital twins are a decision aid, so that in the context of orthopaedic implants the surgeon can select the optimal combination of implant and positioning to achieve the best outcome for that patient [136]. This data then becomes an input for intra‐operative navigation, robotics, or augmented reality technology by surgeons to hit the ideal target identified by the modelling software [137, 138, 139]. Moving beyond selecting implant position based on simple measurements of patient alignment and surgeon preference, this type of patient‐specific modelling will combine data about the individual patient with information from the outcomes of similar patients who have undergone the surgery previously and have had better or worse outcomes. This demonstrates how the future of orthopaedic imaging is moving beyond simply creating an image for interpretation by a clinician to the implementation of the image into a quantitative digital pipeline that improves decision‐making for enhanced patient care. However, to implement this type of modelling to its fullest potential, robust validation and economic analyses are necessary, as are improvements to data harmonization and sharing across centres.

Aside from developing digital tools that use images, there is still opportunity in developing further sophisticated imaging technology and implementing new applications for the technology. The clinical translation of new imaging developments is directly impacted by the accessibility of the modality. Developments in readily available modalities like X‐ray and ultrasound can be implemented immediately. Similarly, advances in CT and MRI, which are commonplace in most hospitals, can also be translated quickly. In contrast, promising new applications for PET and specialized versions of other modalities, such as weight‐bearing CT and low‐field MRI, face longer translational timelines. The more expensive modalities like MRI, PET/MRI, and PET/CT are useful for implant research and in tertiary care settings treating the most difficult implant complications. However, the increasing use of ambulatory surgical centres to perform implant surgeries presents opportunities for less expensive but no less sophisticated modalities like weight‐bearing CT, fluoroscopy, ultrasound, and low field MRI that are well suited for a low‐resource clinic setting. Despite orthopaedic imaging tracing its roots to Roentgen's discovery of x‐rays in 1895, there remains ample opportunity 130 years later for innovation in orthopaedic imaging to improve how orthopaedic implant technology is developed, implanted, and evaluated.
